# New Insights from IGF-IR Stimulating Activity Analyses: Pathological Considerations †

**DOI:** 10.3390/cells9040862

**Published:** 2020-04-02

**Authors:** Joseph A.M.J.L. Janssen

**Affiliations:** Department of Internal Medicine, Erasmus MC, Room Rg 527 Dr. Molewaterplein 40, 3015 GD Rotterdam, The Netherlands; j.a.m.j.l.janssen@erasmusmc.nl; Tel.: +31-06-5003-2421 or +31-10-7040704

**Keywords:** IGF-I, IGF-II, insulin, IGF-IR, IRs, tyrosine kinase receptor, GPCRs, hybrids, phosphorylation, G-proteins, β-arrestins, functional RTK/GPCR hybrid, nuclear translocation

## Abstract

Insulin-like growth factor-I (IGF-I) and insulin-like growth factor-II (IGF-II) play a crucial factor in the growth, differentiation and survival of cells in health and disease. IGF-I and IGF-II primarily activate the IGF-I receptor (IGF-IR), which is present on the cell surface. Activation of the IGF-IR stimulates multiple pathways which finally results in multiple biological effects in a variety of tissues and cells. In addition, activation of the IGF-IR has been found to be essential for the growth of cancers. The conventional view in the past was that the IGF-IR was exclusively a tyrosine kinase receptor and that phosphorylation of tyrosine residues, after binding of IGF-I to the IGF-IR, started a cascade of post-receptor events. Recent research has shown that this view was too simplistic. It has been found that the IGF-IR also has kinase-independent functions and may even emit signals in the unoccupied state through some yet-to-be-defined non-canonical pathways. The IGF-IR may further form hybrids with the insulin receptors but also with receptor tyrosine kinases (RTKs) outside the insulin-IGF system. In addition, the IGF-IR has extensive cross-talk with many other receptor tyrosine kinases and their downstream effectors. Moreover, there is now emerging evidence that the IGF-IR utilizes parts of the G-protein coupled receptor (GPCR) pathways: the IGF-IR can be considered as a functional RTK/GPCR hybrid, which integrates the kinase signaling with some IGF-IR mediated canonical GPCR characteristics. Like the classical GPCRs the IGF-IR can also show homologous and heterologous desensitization. Recently, it has been found that after activation by a ligand, the IGF-IR may be translocated into the nucleus and function as a transcriptional cofactor. Thus, in recent years, it has become clear that the IGF-IR signaling pathways are much more complex than first thought. Therefore a big challenge for the (near) future will be how all the new knowledge about IGF-IR signaling can be translated into the clinical practice and improve diagnosis and treatment of diseases.

## 1. Introduction

The insulin-IGF system is formed by insulin, two insulin-like growth factors (IGF-I and IGF-II), four cell-membrane receptors (insulin receptor-A (IR-A), insulin receptor-B (IR-B), insulin-like growth factor-I receptor (IGF-IR) and insulin-like growth factor receptor-II (IGF-II-R)) and six IGF-binding proteins (IGFBP-1-6), several IGFBP- related proteins and IGFBP proteases [1,2,3,4]. All IGFBPs can bind both IGF-I and IGF-II (however with different binding affinity for some) [5]. Only the unbound forms of IGFs are thought to interact with the IGF-IR and the IGF-II receptor [6].

The IGF-I gene comprises a highly conserved sequence and contains six exons, which give rise to heterogeneous mRNA transcripts by a combination of multiple transcription initiation sites and alternative splicing [7]. These multiple transcripts code in humans for different precursor IGF-I polypeptides, namely the IGF-IEa, IGF-IEb and IGF-IEc isoforms, which also undergo posttranslational modifications, such as proteolytic processing and glycosylation [7]. Differential biological activities have been reported for the different IGF-I isoforms and thus both common and unique or complementary pathways exist for the IGF-I isoforms to promote biological effects [7].

As IGFs and insulin as well as the IGF-IR and the IRs share high sequence homology, they are able to bind and activate each other’s cognate receptors but with considerably lower avidity. The IGF-IR can bind IGF-I and IGF-II with equally high affinity (10^−10^ M) whereas its affinity for insulin (10^−8^ M) is much lower [8]. In the past it was thought that the IGFs and the IGF-IR predominantly mediated growth-promoting effects whereas insulin and the IRs predominantly mediated metabolic effects [9,10]. However, in certain circumstances IGF-I and insulin can mediate very similar responses [11]. Nevertheless, IGF-I and IGF-II play a crucial factor in the regulation of growth, proliferation, differentiation, migration and survival of cells. In addition, activation of IGF-IR and its intracellular pathways has been found to be essential for growth of cancers [12].

IGF-IIR regulates the amount of circulating and tissue IGF-II by transporting IGF-II into the cell and degrading it [13]. IGF-II can also bind to the IGF-IR with high affinity [13].

Due to alternative splicing of exon 11 of the IR gene, two IR transcripts are generated in the human body: IR-A (lacking exon 11) and IR-B (with exon 11) [14,15,16]. The IR-A is predominantly expressed in fetal tissues, the central nervous system, hematopoietic cells and in cancer tissues [14]. The IR-B is predominantly expressed in the liver, muscles and fat cells, the major target tissues for the metabolic effects of insulin [14]. The binding of insulin to IR-B will mainly induce metabolic effects (glucose uptake, glycogen synthesis, glycolysis and fatty acid synthesis) in liver, muscles and adipocytes [14]. Binding of insulin to the IR-A will predominantly induce growth-promoting effects in fetal tissues and tumors. In contrast to IR-B, the IR-A may also bind IGF-II with high affinity and thereby stimulate growth-promoting effects [14].

Although the liver is the main producer of the circulating IGFs, the IGFs are synthesized in almost all tissues of the body [3].

The Insulin-like Growth Factor Binding Proteins (IGFBPs) are a family of six proteins with high affinity for the IGFs. They are widely expressed in most tissues and are flexible endocrine and autocrine/paracrine regulators of IGF activity, which is essential for this important physiological system [3]. IGFBPs may affect cells in both an IGF-dependent and -independent manner [5]. Although IGFBPs often inhibit IGF actions in many circumstances, in some conditions they may also potentiate IGF actions [5].

## 2. IGF-I and the IGF-I Receptor

The IGF-IR is displayed on the cell surface and expressed by nearly all human tissues and cell types [5,9,17]. Surface density of the IGF-IR represents an important determinant of the magnitude of responses to IGF-I and the signaling pattern it provokes [18]. The IGF-IR is a heterotetrameric transmembrane protein composed of two alpha and two beta subunits which are linked by disulfide bonds [19,20]. The beta subunit of the IGF-IR consists of a short extracellular domain which is involved in linkage to the alpha subunits, a transmembrane domain and a cytoplasmic domain containing tyrosine kinase activity [21]. The beta subunit contains a consensus ATP-binding sequence and multiple tyrosine residues that are phosphorylated following ligand binding to the alpha subunit [21]. Binding of IGF-I or another ligand to the alpha subunit of the IGF-IR, induces a closer proximity of regions within the transmembrane domain resulting in autophosphorylation of three intracellular tyrosine residues (Tyr^1131^, Tyr^1135^, and Tyr^1136^) within the beta subunit [21,22,23].

**Figure 1:**
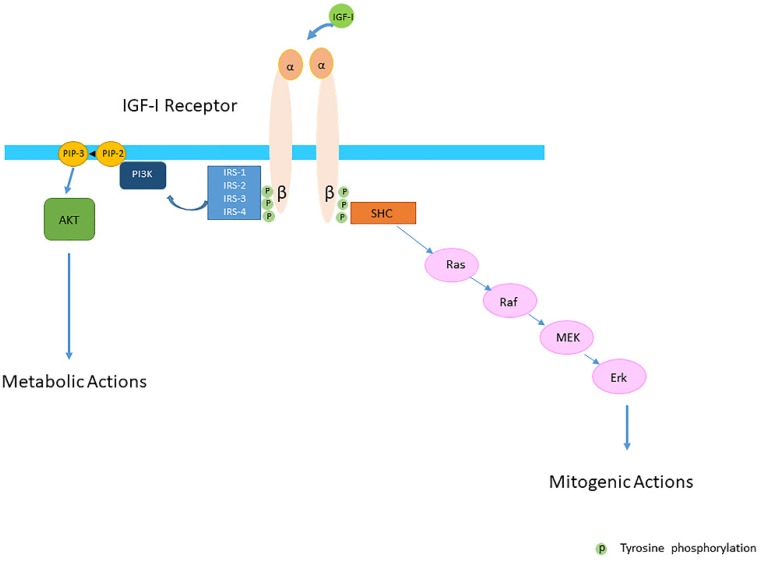
The Insulin-like Growth Factor-I (IGF-IR) is a transmembrane protein composed of two alpha (α) and two beta (β) subunits. The conventional view was that the IGF-IR was exclusively a tyrosine kinase receptor and that the binding of IGF-I to the IGF-IR started the intracellular downstream signal cascade. In this model IGF-I or IGF-II binding to the IGF-IR promotes tyrosine kinase activity and autophosporylation of the beta subunit of the IGF-IR. Intracellularly the activated IGF-IR receptor recruits phosphorylated substrates Insulin receptor substrates (IRSs) and SH2 containing collagen-related proteins (SHC). Tyrosine phosphorylation of IRSs and SHC proteins induces downstream signaling activation through the PI3K-AKT and Ras/Raf/MEK/Erk pathways. It was further thought that activation of the PI3K-AKT pathway had predominantly metabolic effects whereas activation of the Ras/Raf/MEK/Erk pathway had predominantly mitogenic effects.

The conventional view was that the IGF-IR was exclusively a tyrosine kinase receptor and that the binding of IGF-I to the IGF-IR was essential to start the intracellular downstream signal cascade (Figure 1). In this model, the activated receptor recruited and phosphorylated intracellularly substrates as the insulin receptor substrate proteins (IRSs) and SH2 containing collagen-related proteins (SHC) (Figure 1). Tyrosine phosphorylation of the IRSs in turn activated then the phosphatidylinositol 3-kinase (PI3K-Akt) pathway and its various biological responses, while tyrosine phosphorylation of SHC induced downstream signaling activation through the Ras/Raf/MEK/Erk pathway [24,25] (Figure 1).

## 3. The IGF-IR and Endocytosis

Many signaling receptors internalize via clathrin-coated pits [26]. Endocytosis of signaling receptors is widely recognized to confer control on cellular signaling responsiveness [27]. Ligand-induced activation typically increases receptor endocytic rate, and internalized receptors engage molecular sorting machineries that specify subsequent transport via divergent lysosomal and recycling routes [27]. These events, in turn, determine the degree to which cellular ligand responsiveness is attenuated (“down-regulated”) or sustained (“re-sensitized”) under conditions of prolonged or repeated ligand exposure [27].

**Figure 2:**
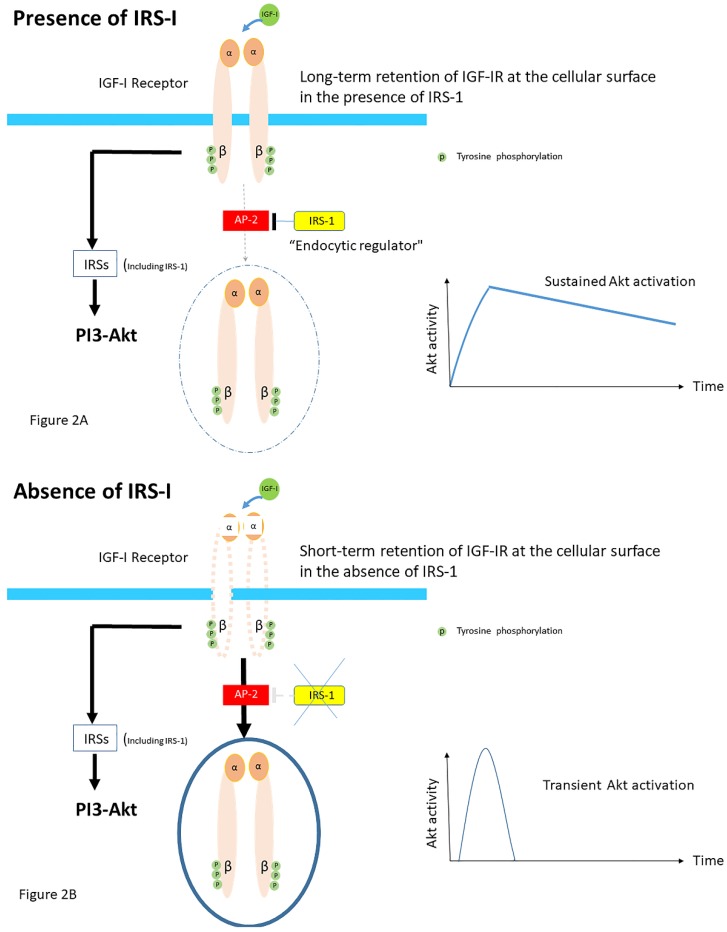
Proposed role of Insulin receptor substrate-1 (IRS-1). IRS-1 modulates how long ligand-activated IGF-IR remains at the cell surface before undergoing endocytosis in mammalian cells. IRS-1 interacts with the clathrin adaptor complex AP2. (**A**) In the presence of the IRS-1/AP2-complex in the cell IGF-IR endocytosis after the ligand stimulation is delayed. Mechanistically, IRS-1 inhibits the recruitment of IGF-IR into clathrin-coated structures; for this reason, IGF-IR avoids rapid endocytosis and prolongs its activity on the cell surface and this results in sustained activation of the AKT pathway. (**B**) In absence of IRS-1/AP2- complex in the cell, there is only short-term retention of the IGF-IR at the cell surface and IGF-IR endocytosis is accelerated. This results in a transient activation of the AKT pathway (Modified from Yoneyama et al. IRS-1 acts as an endocytic regulator of IGF-I receptor to facilitate sustained IGF signaling. eLIFE, 2018; 7. pii: e32893).

The molecular basis for the close interactions between IGF-IR endocytosis and its signaling components is still poorly understood. Recently, it has been suggested that the ability of insulin receptor substrate-1 (IRS-1) to interact with the clathrin adapter protein AP2, which is essential for endocytosis, plays an important role in IGF-IR internalization [28]. Overexpression of IRS-I resulted in the accumulation of activated IGF-IR at the cellular membrane [28]. Conversely, knockdown of IRS-I induced faster internalization of IGF-IRs [28]. These data suggest that IRS-1 inhibits the recruitment of IGF-IR into clathrin-coated structures; the ability of IRS-I to bind to AP-2 avoids rapid endocytosis of the IGF-IR and prolongs its activity at the cell surface in HEK293T cells [28] (Figure 2A). In contrast, accelerating IGF-IR endocytosis via IRS-1 depletion induces the shift from sustained to transient Akt signaling [28] (Figure 2B). Thus, independent of its classic role as an adaptor in IGF-I receptor signaling, IRS-1 has a role as an endocytic regulator of IGF-I receptor that ensures sustained IGF bioactivity, while IRS-1 degradation could be a trigger to internalize the IGF-IR [29].

For the IGF-IR, ubiquitination also increases upon ligand binding [30]. The IGF-IR has been demonstrated to be a substrate for three ubiquitin ligases: Mdm2, (in human malignant melanoma cells), c-Cbl (HEK293 cells and human osteosarcoma cell lines U2OS and SAOS2) and Nedd4 (in mouse embryo fibroblasts). [30,31]. Mdm2 was originally described to control IGF-IR ubiquitination and thereby causing its degradation by the proteasome system [32]. Subsequently β-arrestins, known to be involved in the regulation of G-protein-coupled receptors (GPCRs), have also been identified as adaptor proteins to bring the oncoprotein Mdm2 to the IGF-IR in mouse embryo fibroblasts [33,34]. In addition, while removing the IGF-IR from the cell surface and inhibiting the “classical” kinase signaling pathway, β-arrestins may redirect the signaling wave through ERK [33] Ubiquitination may thus induce receptor internalization and degradation, but also enhance IGF-IR signaling [35] (which will be further addressed in the paragraph “the complexity of the post-receptor IGF-IR/IR pathways” below).

## 4. Structural Differences and Overlap between the IGF-IR and the IRs

It is hypothesized that the IGF-IR and IRs are created by gene duplication of common precursor receptor molecule [36]. Due to structural and functional homology, IGF-I and insulin can bind to (and activate) both IGF-IR and the IRs, as discussed above [37]. IGF-IR and IRs show 48% amino acid sequence homology [20]. Structural differences between the beta-subunit and kinase domains of the IGF-IR and the IRs leading to differences in substrate interactions may be (partly) responsible for IGF-I and insulin specificity as has been found in various cell types (rat-1 fibroblasts, murine skin keratinocytes and in NIH-3T3-fibroblasts) [38]. However, the signal transduction by the receptors may not be limited to its activation at the cell surface [39].

In addition to signaling through the classical tyrosine kinase pathways, it has been found that the IGF-IRs and IRs (in cells derived from C57Bl/6 mice) can emit signals in the unoccupied state through some yet-to-be-defined non-canonical pathways [40]. Boucher et al. demonstrated that cells lacking the IGF-IR and IR exhibit a major decrease in expression of multiple imprinted genes and microRNAs [40].

Although the IGF-IR and IRs have both distinct and overlapping functions, it has been suggested that in vivo specificity of the IGFs and insulin are at least in part reflected by the timing of the expression of the IGF-IR and IRs in target tissues in combination with ligand concentration and availability [41]. It has been further suggested that IGF-I and the IRs act as identical portals for the regulation of gene expression and that the differences between IGF-I and insulin effects are due to a modulation of the amplitude of the signal created by the specific ligand receptor interaction [41].

## 5. The IGF-IR and the IRs May Form Hybrids in the Human Body

**Figure 3:**
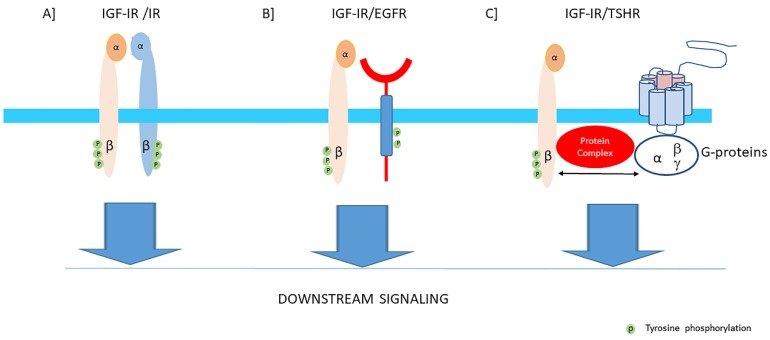
The IGF-I receptor may form hybrids with the insulin receptor, many other tyrosine kinase receptors outside the insulin-IGF system and G-protein coupled receptors. The figure shows three examples: (**A**) Hybrids may be formed consisting of an alpha-beta subunit of the IGF-IR linked by disulfide bonds to an alpha-beta subunit of the IR. Downstream signaling of both receptors converge via the canonical PI3K-Akt and ERK signaling pathways. Most functional studies have found that hybrid receptors behave more like IGF-IRs than IRs (See also text). (**B**) Hybrids may be formed consisting of an alpha-beta subunit of the IGF-IR linked and a monomer of the epidermal growth factor receptor (EGFR) which is also a tyrosine kinase receptor. Downstream signaling of both receptors converge via the canonical PI3K-Akt and ERK signaling pathways. Therefore, inhibition of one receptor of these hybrids may shift the signaling pathway in favor of the other available counterpart receptor. (**C**) The Thyroid Stimulating Hormone Receptor (TSH receptor), a typical G-protein coupled receptor, may form functional hybrids with the IGF-IR in the cellular membrane by forming a common protein complex. Bidirectional crosstalk between the IGF-IR and TSHR has been demonstrated. Stimulation of the IGF-IR by IGF-I/IGF-IR agonists may trigger the classical signaling pathway of the IGF-IR, leading to downstream kinase-cascade signaling activation. In addition, stimulation of the IGF-IR by IGF-I/IGF-IR agonists may also utilize components of G-protein coupled receptor (GPCR) signaling and activate pathways conventionally used by TSHR.

In cells and tissues where both significant levels of the IGF-IRs and IRs are present, hybrids may be formed consisting of an alpha-beta subunit of the IGF-IR linked by disulfide bonds to an alpha-beta subunit of the IR [42] (Figure 3A). They are formed in the endoplasmic reticulum before they reach the cell surface [43]. Two splice variants of the hybrid receptors exist for the IR because the IR is expressed (as above discussed) either with (IR-B) or without 12 amino acids encoded by exon 11 (IR-A) [44]. Thus both IR-A/IGF-IR (Hybrid A) and IR-B/IGF-IR (Hybrid B) receptors can be formed. Although the biological functions of these hybrid receptors is still unclear, it has been suggested that hybrid receptors may play a role in the overlapping functions of IGF-I and insulin [21]. Several studies (in baby hamster kidney cells, NIH3T3 cells overexpressing IGFR and CHO cells overexpressing IR-B) have suggested that cells by increasing the relative expression level of the IGF-IR above that of the IR lose their insulin sensitivity because hybrid receptors bind insulin with low affinity [45,46]. In addition, binding of insulin to the alpha-beta subunit of the IR which is part of a hybrid, may result in autophosphorylation of its ow beta subunit and, following transphosphorylation of the beta subunit of the IGF-IR, may result in a signal for growth, [9]. In contrast, when IGF-I binds to the alpha-beta subunit of the IGF-IR, this may activate the beta subunit of the IRs by the same mechanisms and thereby activate growth (IR-A) or metabolism (IR-B) [9]. Although this latter mechanism could explain why hybrids may stimulate metabolic functions when stimulated by IGF-I, most functional studies have found that hybrid receptors behave more like IGF-IRs than IRs [47]. It has been hypothesized that this prioritization of hybrid receptors to IGF-I results from the ability of IGF-I to activate monomeric IGF-IR whereas, in contrast, dimerization of the IR has been considered necessary to induce a response to insulin [45].

In cells and tissues where both significant levels of the IGF-IRs and IRs are present, hybrids may be formed consisting of an alpha-beta subunit of the IGF-IR linked by disulfide bonds to an alpha-beta subunit of the IR [42] (Figure 3A). They are formed in the endoplasmic reticulum before they reach the cell surface [43]. Two splice variants of the hybrid receptors exist for the IR because the IR is expressed (as above discussed) either with (IR-B) or without 12 amino acids encoded by exon 11 (IR-A) [44]. Thus both IR-A/IGF-IR (Hybrid A) and IR-B/IGF-IR (Hybrid B) receptors can be formed. Although the biological functions of these hybrid receptors is still unclear, it has been suggested that hybrid receptors may play a role in the overlapping functions of IGF-I and insulin [21]. Several studies (in baby hamster kidney cells, NIH3T3 cells overexpressing IGFR and CHO cells overexpressing IR-B) have suggested that cells by increasing the relative expression level of the IGF-IR above that of the IR lose their insulin sensitivity because hybrid receptors bind insulin with low affinity [45,46]. In addition, binding of insulin to the alpha-beta subunit of the IR which is part of a hybrid, may result in autophosphorylation of its ow beta subunit and, following transphosphorylation of the beta subunit of the IGF-IR, may result in a signal for growth, [9]. In contrast, when IGF-I binds to the alpha-beta subunit of the IGF-IR, this may activate the beta subunit of the IRs by the same mechanisms and thereby activate growth (IR-A) or metabolism (IR-B) [9]. Although this latter mechanism could explain why hybrids may stimulate metabolic functions when stimulated by IGF-I, most functional studies have found that hybrid receptors behave more like IGF-IRs than IRs [47]. It has been hypothesized that this prioritization of hybrid receptors to IGF-I results from the ability of IGF-I to activate monomeric IGF-IR whereas, in contrast, dimerization of the IR has been considered necessary to induce a response to insulin [45].

It has been further hypothesized that IGF-IR/IR hybrids may affect tumor biology [48]. Specific downregulation of the IGF-IR by agents solely targeting the IGF-IR diminishes hybrid formation and this thereby enhances holo-IR formation [48]. An enhanced holo-IR formation results in an increase of insulin sensitivity [48]. As the IR, especially the IR-A, may also activate (post-receptor) signaling pathways involved in growth similar to the IGF-IR, the development of agents simultaneous targeting both IR-A and IGF-IR may be necessary to disrupt the malignant phenotype of cancers cells that are influenced by actions of the insulin-IGF system [48].

The IGF-IR may also heterodimerize with receptor tyrosine kinases (RTKs) outside the insulin-IGF system [49]. Heterodimerization of the IGF-IR with the EGFR is well-established [50] (Figure 3B). Downstream signaling of both receptors converge via the canonical PI3K-Akt and ERK signaling pathways. Therefore inhibition of one receptor in these hybrids may shift the signaling pathway in favor of the other available counterpart receptor [49,51,52]. Thus the compensatory signaling may be bidirectional [53]. Moreover, evidence exists that IGF-IR can activate independently downstream EGFR pathways and this may subsequently result in EFGR tyrosine kinase inhibitor (TKI) resistance [52]. The IGF-IR signaling pathway shows also cross-talk with the growth hormone receptor (GHR), thyroid stimulating hormone receptor (TSHR) (Figure 3C), estrogen receptor (ER), androgen receptor (AR) and human epidermal growth factor receptor 2 (HER-2) signaling pathways [54,55,56,57,58,59].

## 6. The Functional Relationship between Insulin/IGF Signaling and Discoidin Domain Receptors

In addition to its canonical role as a collagen receptor, it has recently been suggested that the discoidin domain receptor-1 (DDR1), a tyrosine kinase receptor, plays an important role in the regulation of the insulin-IGF system [60]. In contrast to most other RTKs, DDR1 is not activated by soluble growth factors but instead by various type of collagens [60]. While most other RTKs are fully activated in minutes, maximal activation of DDR1 occurs several hours after initial stimulation with collagen [60]. The DDR1 and the insulin-IGF system are linked by a feed-forward mechanism by which insulin and the IGFs induce DDR1 upregulation which in turn enhances expression and activity of the IRs and the IGF-IR [60]. The mechanisms by which DDR1 may affect downstream signaling of the IRs and IGF-IR are as yet not fully understood. It has been found that increasing DDR1 expression favors the expression of the more mitogenic IR-A isoform over the metabolic IR-B isoform and thus one of the functional consequences of this DDR1 upregulation may be increased IGF-II signaling through the IR-A [60]. This in turn may favor dedifferentiation and stem-like features [60]. It has been further hypothesized that inhibition of DDR1 may be a way to downregulate the tumor-inducing actions of the insulin/IGF system in human cancer cells while simultaneously inducing differentiation of cells without affecting the IR-B mediated metabolic actions [60]. In favor of this latter hypothesis, in vitro DDR1 silencing or downregulation blocked the IGF-2/IR-A autocrine loop in poorly differentiated thyroid cancer cells and induced cellular differentiation [61]. Although at present no clinical studies have shown that this strategy provides any clinical benefits for patients with tumors overexpressing the DRR1 and the insulin-IGF-I system, these results may help to develop novel therapeutic approaches for cancer.

## 7. The Complexity of the Post-Receptor IGF-IR/IR Pathways

**Figure 4:**
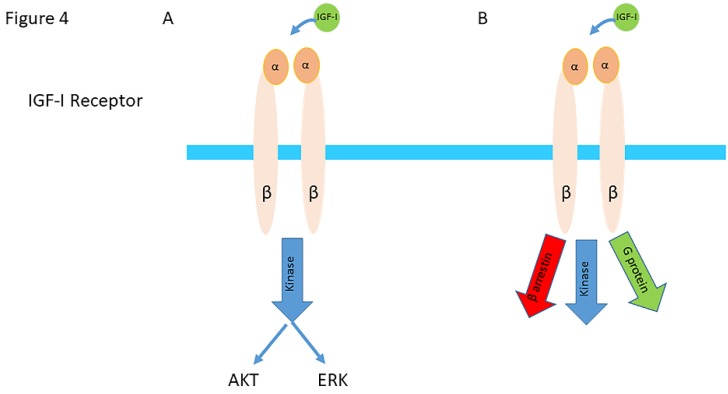
(**A**) In the classical model IGF-IR activation was triggered exclusively by ligand binding and signaling was exclusively mediated by a kinase cascade through phosphorylation. The ligand-activated IGF-IR was thought to lead to a balanced stimulation of the AKT/ERK pathways. (Abbreviation AKT= protein kinase B; ERK= extracellular signal –regulated kinase) (**B**) In the current model, binding of a ligand to the IGF-IR results not only in stimulating of the kinase cascade through phosphorylation of IRS-1, PI3K and AKT but also in activation and signaling by G-proteins and β-arrestins, as well as desensitization and internalization by β-arrestins. In this model ligand binding results in a balanced activation and signaling of the kinase cascade, G-proteins and β-arrestins, as well as desensitization and internalization by β-arrestins. (Modified from Girnita et al. Something old, something new and something borrowed: emerging paradigm of insulin-like growth factor type 1 receptor (IGF-1R) signaling regulation. Cell Mol Life Sci. 2014; 71:2403-27).

In recent years, it has been become clear that the downstream complexity of the IGF-IR/IR pathways was grossly underestimated in the past. In this section we will discuss some important novel insights regarding this complexity. In the classical model, as discussed above, the IGF-IR was traditionally described as a tyrosine kinase receptor with an ON/OFF (active/inactive) system. In this system IGF-I binding to the IGF-IR stabilized the ON state (active) and this exclusively mediated kinase-dependent signaling activation of both the PI3-AKT and ERK pathways [62,63] (Figure 4A). Ubiquitin-mediated receptor downregulation and degradation was originally described as a response to ligand/receptor interaction and thus inseparable from kinase signaling activation [62].

Almost 25 years ago, a study from Nobel Prize winner Dr. Robert Lefkowitz’s laboratory reported that IGF-IR-dependent activation of the Mitogen-Activated Protein Kinase (MAPK) signaling pathway was inhibited by the Gαi-inhibitor pertussis toxin [64]. The last years there is emerging evidence that many RTKs can also utilize heterotetrameric G proteins to subserve some of their biological actions [65,66,67]. Recently, more extensive evidence has been published that the IGF-IR and IRs are also engaged in in G-protein coupled receptor (GPCR) signaling [62,63,65,66,67]. As GPCR phosphorylation by GPCR-kinases (GRKs) governs interactions of the receptors with β-arrestins, Zheng et al. investigated the regulatory roles of the four widely expressed GRKs on IGF-IR signaling/degradation [68]. They found that lowering GRK5/6 abolished IGF-I-mediated ERK and AKT activation, whereas GRK2 inhibition increased ERK activation and partially inhibited AKT signaling [68]. In addition, β-arrestin-mediated ERK signaling was enhanced by overexpression of GRK6 and diminished by GRK2. Similarly, they demonstrated opposing effects of GRK2 and -6 on IGF-IR degradation: GRK2 decreased whereas GRK6 enhanced ligand-induced degradation [68]. GRK2 and GRK6 co-immunoprecipitated with IGF-IR and increased IGF-IR serine phosphorylation, promoting β-arrestin1 association. Thus this study demonstrated distinct roles for GRK2 and GRK6 in IGF-IR signaling through β-arrestin binding with divergent functional outcomes [68].

**Figure 5:**
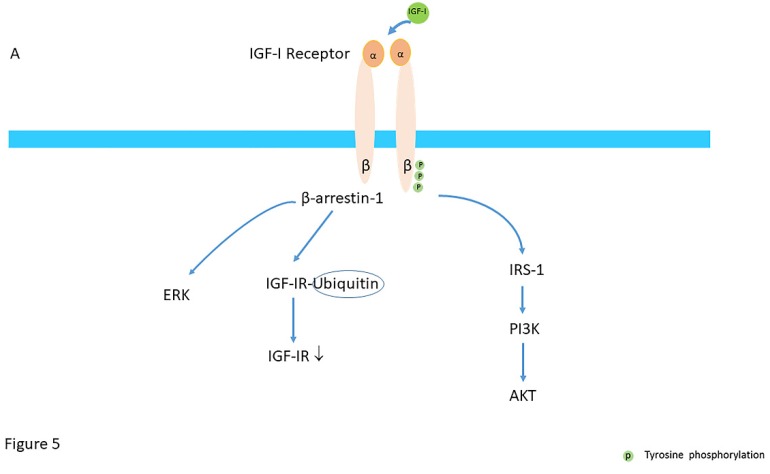

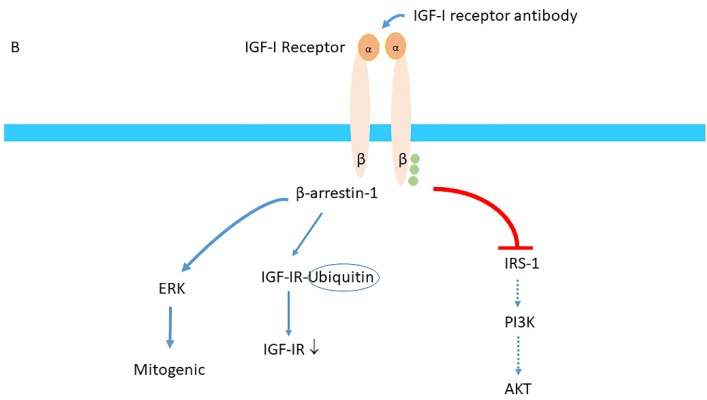
(**A**) Mechanisms of balanced agonism; activation of the IGF-IR stimulates not only the AKT pathway by phosphorylation of IRS-I and PI3K, but in addition, stimulates the β-arrestin-1 (β-arr1) pathway which leads to proteasomal degradation of the IGF-IR through an ubiquitin (Ub)-mediated mechanism and ERK activation. (**B**) Beta-arrestin-1 biased agonism. Binding of monoclonal (blocking) antibodies directed against the IGF-IR block the kinase cascade pathway (by blocking phosphorylation of IRS-1, PI3K and AKT) but simultaneously activate the β-arrestin-1 (β-arr1) pathway which induces enhanced IGF-IR receptor internalization (ubiquitination) and activation of ERK signaling pathway. (Modified from Salisbury & Tomblin. Insulin/Insulin-like growth factors in cancer: new roles for the aryl hydrocarbon receptor, tumor resistance mechanisms, and new blocking strategies. Front Endocrinol (Lausanne), 2015; 6:12).

Based on the insight that IGF-IR may also “borrow” components of GPCR signaling, including β-arrestins and G-protein-coupled-receptor kinases (GRKs), a new paradigm has emerged for the IGF-IR [62]. In this new paradigm, the IGF-IR is considered to be a functional RTK/GPCR hybrid, which integrates the kinase signaling with some IGF-IR mediated canonical GPCR characteristics [62]. Binding of IGF-I to the IGF-IR thus not only leads to balanced phosphorylation-dependent Akt/ERK signaling intracellularly, but results simultaneously also in activation of signaling by G-proteins and β-arrestins [62] (Figure 4B and Figure 5A).

The IRs have been shown also to interact with G-proteins and β-arrestin-1 [69]. However, the IGF-IR and IRs engage in different G-proteins for downstream signaling [65]. This possibly provides a mechanism that is responsible for the signaling specificity of these two receptors [65].

Also another new paradigm, the paradigm of biased signaling, has been proposed for IGF-IR and IR signaling [67,70]. The paradigm of biased signaling also originates from the GPCR signaling field [71]. The regulatory process which was discovered as the means by which classical GPCRs “desensitized“ or tuned off, has been also found to be active for the IGF-IR [71]. In this paradigm the ligand is biased towards a specific signaling i.e., the signal mediated by binding of a ligand to a receptor is no longer balanced but the ligand elicits one response of the ligand over another compared with the classical ligand [53,63]. The IGF-IR has been extensively studied as an anti-cancer target However, monotherapy trials with IGF-IR targeted antibodies, have, overall, been very disappointing [12]. The anti-IGF-IR antibody Figitumumab (CP-751,871; CP) was designed as an antagonist to prevent ligand-receptor interaction [72]. Although it was found that CP blocked the kinase cascade pathway (by blocking phosphorylation of IRS-1, PI3K and AKT), as with all anti-IGF-IR antibodies, it simultaneously induced IGF-IR/β-arrestin-1 association with dual functional outcome: receptor ubiquitination and degradation and decrease in cell viability and β-arrestin-1-dependent ERK signaling activation [72].

Thus despite blocking all the “classical” tyrosine kinase-mediated effects of the IGF-IR, a blocking antibody directed against the IGF-IR may function as an IGF-IR/β-arrestin-1/ERK agonist and favor β-arrestin-1/ERK signaling [73] (Figure 5B). Another example of biased signaling of the IGF-IR signaling is mediated by LL-37, a newly recognized bacterial peptide, which after binding to the IGF-IR may function as an IGF-IR agonist by increasing β-arrestin-1 signaling and electively activating the ERK pathway but without affecting simultaneously the PI3K-AKT pathway [74].

One should make a distinction between homologous and heterologous desensitization. Homologous desensitization occurs within a receptor system when it alters its own responsiveness, for example the loss of responsiveness (desensitization) that can occur upon binding of insulin to the IR [75]. It is considered to limit or restrain a cell’s responses to certain stimuli; it leaves a cell (transiently) less- or unresponsive to a ligand that activates the desensitized receptor but not to ligands that activate other receptors. In contrast, in heterologous desensitization the responsiveness of one receptor system is regulated (positively or negatively) by activation of another receptor system (i.e., “cross-talk”) [75]. For example, insulin after binding to the IR may induce heterologous desensitization of the signaling of the IGF-IR by downregulating β-arrestin-1 and inhibiting of IGF-I-stimulated MAP kinase phosphorylation [76]. However, it has been found that this latter effect could be substantially rescued by ectopic expression of wild-type β-arrestin-1, consistent with the view that the decrease in cellular β-arrestin-1 content is a major mechanism for the observed desensitization effects of insulin on IGF-IR mediated signaling [76].

## 8. The Nuclear Translocation of the IGF-IR and IRs and Its Significance

The IGF-IR and IRs not only function at the cell surface. When after binding of IGF-I to the IGF-IR and the IGF-I/IGF-IR complex has been internalized into the cell, there are three potential outcomes for the internalized IGF-IR: it can go back to the cellular surface, it can be degraded or it can go to the nucleus [77].

It has been documented that both the IGF-IR and IRs can be translocated to the nucleus [78,79]. Nuclear transport of IGF-IR is enhanced by IGF-I and IGF-II but only modestly by insulin [79]. This transport correlated directly with the magnitude of ligand-induced receptor phosphorylation of the IGF-IR with these ligands [79]. In addition, it has been found that ligand-mediated phosphorylation of the IGF-IR is essential for nuclear trafficking [78].

IGF-IR nuclear import and chromatin binding can be blocked by an IGF-IR kinase inhibitor, indicating that indeed IGF-IR kinase activity is required for the IGF-IR to enter the nucleus [79].

The IGF-IR can undergo both caveolin- and clathrin mediated endocytosis [80,81]. Consistent with clathrin-mediated endocytosis, nuclear IGF-IR translocation can be blocked by the inhibitors of clathrin-dependent endocytosis (dansylcadaverine and the dynamin-1 inhibitor dynasore), but not by caveolin-1 depletion [79]. Nevertheless, the exact mechanisms responsible for nuclear import of the IGF-IR and IRs are still unclear [78]. Sehat et al. found that the α subunit (native size, 120 kD) together with the β subunit (native size, 95 kD) was present in the nuclear fraction, suggesting that nuclear IGF-IR was an intact receptor [82]. Data of Aleksic et al. also suggested that full-length IGF-IR translocates to the nucleus [79].

**Figure 6:**
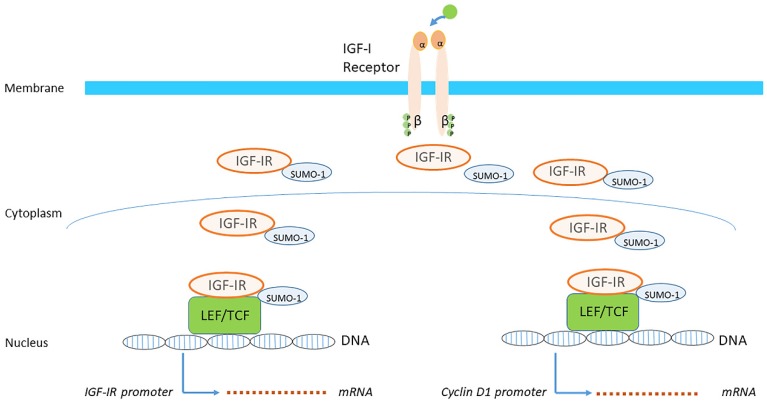
Studies have confirmed that ligand-mediated phosphorylation of the IGF-IR is essential for nuclear trafficking. Following binding of IGF-I to the IGF-IR at the cell surface, the IGF-IR is transported into the cell and further translocated from the cytoplasm into the nucleus. SUMOylation (SUMO-1) in the cytoplasm of the IGF-IR also plays a crucial role in the nuclear translocation of the IGF-IR from the cytoplasm. When the SUMOylated IGF-IR translocates to the nucleus, it is thought to be involved in the transcriptional enhancement of specific target genes. Nuclear IGF-IR is able to autoregulate expression of its own gene leading to an increase in IGF-IR promoter activity and IGF-IR expression (left). Nuclear IGF-IR may also bind to Cyclin D1 (and additional) promoters with ensuing target gene activation (right). (Modified from Sarfstein & Werner. Minireview: nuclear insulin and insulin-like growth factor-1 receptors: a novel paradigm in signal transduction. Endocrinology, 2013; 154:1672-9).

Both the IGF-IR and IRs are present in the perinuclear and nucleolar area of the nucleus in a small ubiquitin-like modifier SUMOylated form [78]. Receptor SUMOylation occurs in a ligand dependent fashion and it has been demonstrated that SUMOylation plays a crucial role in the nuclear translocation of the IGF-IR [82,83] (Figure 6). The SUMO-modified IGF-IR is deSUMOylated after passage across the nuclear membrane [82]. Nuclear IGF-IR binds to enhancer regions and activates transcription [84]. It is able to autoregulate expression of its own gene leading to an increase in IGF-IR promoter activity and IGF-IR expression [78] (Figure 6).

It has been further suggested that nuclear IGF-IR has biological significance in cancer; prognosis was less good and survival was shorter in patients whose tumor showed intense and/or widespread nuclear IGF-IR [79]. It has been reported that nuclear IGF-IR is a feature of pre-invasive lesions and invasive cancers including prostate, renal and breast cancers, and an association between nuclear IGF-IR and adverse prognosis was identified in renal cancer [79]. Subsequent data did associate nuclear IGF-IR with proliferation, tumorigenicity, resistance to EGFR inhibition and clinical response to therapeutic anti-IGF-IR antibodies, which suggests that IGF-IR nuclear import has biological significance and may contribute directly to IGF-IR function [79,85,86,87,88,89,90].

When the IGF-IR translocates to the nucleus, it is thought to be involved in the transcriptional enhancement of specific target genes [79,82,91]. It has been demonstrated that IGF-IR in the nucleus binds to the transcription factor lymphoid enhancer factor-1 (LEF1), leading to elevated protein levels of cyclin D1 and axin2 [84] (Figure 6). This might be an additional molecular mechanism by which IGF-IR promotes uncontrolled cell proliferation and contribute to the neoplastic transformation of cells [84].

When investigating the impact of IGF-IR levels on IGF-IR biosynthesis in estrogen receptor positive (ER+) and estrogen receptor depleted (ER-) breast cancer cells, it was found that in ER+ cell and ER- cells regulation of the IGF-IR gene and IGF-IR protein differed at the level of transcription; the IGF-IR protein was able to stimulate IGF-IR gene expression in ER- cells but not in ER+ cells [92]. Similarly to the IGF-IR, it was found that the IR was also translocated to the nucleus and to bind to the IGF-IR promoter. However, this was only observed in ER- cells but not in ER+ cells [92].

In addition, it has been found that transcription factors IGF-IR and IR display diametrically opposite activities in the context of IGF-IR gene regulation; in contrast to the IGF-IR, IR inhibited IGF-IR promoter activity [92]. Thus nuclear IGF-IR acted as a transcriptional activator of its own promoter, while nuclear IR functioned as a negative regulator of IGF-IR promoter activity [92]. Nevertheless, the authors of this latter paper concluded that the clinical implications of their findings—in particular the impact of IGF-IR/IR nuclear localization on targeted therapy—are at present unclear and require further investigation [92].

## 9. Conclusions

Although until recently the conventional view was that phosphorylation of tyrosine residues played a major role in the activation of the IGF-IR and initiated all downstream signaling, there is increasing evidence showing that this view was too simplistic and grossly underestimated the downstream complexity of the IGF-IR pathways. The IGF-IR has not only extensive cross-talk with many other receptors, but that the IGF-IR can be also considered as a functional RTK/GPCR hybrid, which integrates the kinase signaling with some IGF-IR mediated canonical GPCR characteristics. Like classical GPCRs the IGF-IR show homologous and heterologous desensitization. In addition, after activation by a ligand, the IGF-IR signaling can be translocated to the nucleus and function as a transcriptional cofactor. For example nuclear IGF-IR is able to autoregulate expression of its own gene.

Thus, it has become clear in recent years that the IGF-IR signaling pathway is far more complex than previously thought. It contains many points of regulation and shows signal divergence and cross-talk with many other signaling pathways at the receptor and post-receptor level. However, a big challenge for the (near) future will be how all this new knowledge about the IGF-IR signaling pathways can be translated into clinical practice and improve diagnosis and treatment of diseases.
